# Cytotoxic Effects of Benzene Metabolites on Human Sperm Function: An *In Vitro* Study

**DOI:** 10.1155/2013/397524

**Published:** 2013-12-12

**Authors:** Priyanka Mandani, Ketki Desai, Hyacinth Highland

**Affiliations:** Department of Human Genetics, Biomedical Technology and Zoology, Gujarat University, Ahmedabad, Gujarat 380009, India

## Abstract

In recent years, individuals are rampantly exposed to vapours of benzene, through paint, plastic, petroleum industries, fuel exhaust, and tobacco smoke. Hence the present investigation was directed towards determining the effect of benzene metabolites, namely, phenol-hydroquinone and catechol, on the motility, viability, and nuclear integrity of the human spermatozoa. From the results obtained it was clear that exposure to phenol-hydroquinone caused a significant decline in both, sperm motility and viability. Exposure to a phenol-hydroquinone (Phase I) microenvironment may therefore inhibit metabolically active enzymes, thus impeding ATP production, and in turn lowers sperm motility and viability. In addition, the present study also revealed that both metabolites of benzene caused significant denaturation of sperm nuclear DNA. Hence, exposure to phenol-hydroquinone *in vitro* could have resulted in generation of free radicals and altered membrane function, which is reflected by a decline in the motility, viability, and loss of sperm nuclear DNA integrity. In Phase II, the exposure of human sperm *in vitro* to varied concentrations of catechol caused only insignificant changes in sperm motility and viability as compared to those observed on exposure to phenol-hydroquinone. Hence, exposure to catechol appeared to have less toxic effects than those of phenol-hydroquinone.

## 1. Introduction

Benzene is an important industrial chemical present in petroleum products that is also omnipresent in the environment due to emissions from gasoline and combustion of hydrocarbons and tobacco [1, 2]. Urban populations throughout the world and cigarette smokers are routinely exposed to air concentrations of benzene in the range of 1–20 ppb [3].

Benzene is both exhaled unchanged in the lungs, as well as metabolized in liver and excreted as metabolites in the urine. The first step in benzene metabolism is the formation of benzene oxide, an epoxide, by cytochrome P-450 dependent mixed function oxidases. The epoxide undergoes hydroxylation to phenol which is then excreted as a glucuronidase or sulphate conjugate or converted to hydroquinone and benzoquinone. Phenol, hydroquinone glucuronide, and hydroquinone sulphate serve as markers for this enzymatic pathway. A second pathway involves conversion of benzene oxide to malondialdehyde through an NADPH mediated process, resulting in catechol production through the intermediate benzene glycol [4]. Significant concentrations of the phenolic compounds (phenol, catechol, and hydroquinone) are observed in human urine even in the absence of prominent exposure to benzene and point to background sources, including diet, cigarette smoking, and microbiome [5].

Men exposed to high levels of benzene are more likely to have an abnormal amount of chromosome in their sperm, which impacts fertility and fetal development. With records of inhalation of benzene exhaust for 8-9 hours/day, for prolonged periods of time, accumulation of its metabolic intermediates could manifest toxic influences on sperm function and these could contribute to foetal loss, foetal abnormalities, and infertility. However, little or no research has been carried out in this regard.

Phenol-hydroquinone is the major metabolite after benzene biotransformation in the liver. Phenol can enter the body by 3 ways: during inhalation of air containing phenol (from industries, automobile exhaust, cigarette smoke, and wood burning) and during ingestion, as phenol in food or water (detached phenol in surface waters, rainwaters, sediments, drinking water, groundwater, hazardous water sites, etc.) may also rapidly enter the body through the digestive tract; and a significant amount may enter through skin. Phenol has been evaluated for genotoxicity in both *in vivo* and *in vitro* test systems. Increased chromosomal aberrations have been reported in bone marrow [6] and in spermatocytes [7] from mice treated with phenol.

Catechol is used as an intermediate for the synthesis of pharmaceuticals and agrochemicals and in formulation. It causes acute toxic effects, namely, skin irritation, serious eye damage, allergic reactions, and genetic defects. However, there are no reports in the literature regarding its effects on the sperm. Since the reproductive tract is exposed to metabolites of benzene, namely, phenol-hydroquinone and catechol, it is therefore imperative to evaluate their toxic effects on the sperm.

Since the sperm is a unique cell, easily available, can be cultured *in vitro*, and is sensitive to its microenvironment, it has therefore proved to be a good target cell for cytotoxic assays, in toxicological studies. Hence, the objective of this study was to evaluate the effect of benzene metabolites phenol-hydroquinone and catechol, on human sperm motility, sperm viability, and sperm DNA integrity (*In vitro* analysis).

D'Cruz et al. [8] have demonstrated that human sperm, under the effect of drugs and toxins, show altered motility, often in association with loss of dynein-ATPase activity. Sperm cell motility is one of the most sensitive parameters and is therefore a good marker for sperm cytotoxic effects. Motility is a sensitive indicator of sperm function and is also affected by periods of abstinence, toxic agents, metal ions, alkaloids, drugs, and so forth. Several inorganic ions and molecules that spermatozoa do not normally encounter comprise the most deleterious pollutants, which inhibit sperm motility. The test for sperm viability aids in the inference of the effect of an agent on sperm metabolism and membrane permeability and thus gives a complete assay of cytotoxic effects. Hence, in this study sperm motility and viability have been regarded as reliable indices to evaluate the presence and effect of benzene metabolites.

Oxidative stress during sperm transport through the male reproductive tract is likely the most frequent cause of sperm DNA damage [9, 10]. Hence, evaluation of DNA damage and nuclear integrity is imperative to determine sperm-related cytotoxic effects which could be induced due to the products of benzene biotransformation. The sperm DNA is known to be sensitive to environmental stress and toxicants [11].

Since benzene is a ubiquitous environmental and occupational toxicant, spermatozoa of workers exposed to benzene vapours are therefore subjected to the effects of its metabolites *in vivo*, during their transit. Hence the present study was aimed at evaluating the functional alteration induced in sperm on *in vitro* exposure to the metabolites of benzene, using the sensitive indices of sperm intoxication.

## 2. Materials and Methods

Semen samples for the study were collected from an authorized sperm bank: Indian Spermtech and Semen Bank, Ellis Bridge, Ahmedabad. Only fertile donors' samples were selected. After liquefaction of 20 minutes, the sperm count of the sample was checked by the WHO (2010) protocol and only those samples with sperm count within the normal range (75–110 millions/mL) were taken. The present investigation was carried out in 2 phases.

In the first phase the effect of Phenol-hydroquinone, at varying concentrations, was evaluated on the freshly collected semen sample to determine the effects on the sperm cell.

A stock solution of Phenol-hydroquinone at 1 ppm concentration (prepared in DMEM-F 12 HiMedia) was initially serially diluted with Dulbecco's Modified Eagle Medium Nutrient Mixture F 12-HAM (1 : 1) (HiMedia) and 30 *μ*L, 20 *μ*L, 10 *μ*L, and 5 *μ*L aliquots (each having concentration of 0.3 ppm, 0.2 ppm, 0.1 ppm, and 0.05 ppm, resp.) were taken and incubated with 100 *μ*L of the freshly obtained sample in separate cuvettes. After 1 hour incubation period at 37°C the sperm motility and viability were checked for each aliquot after which smears were prepared and fixed for sperm nuclear integrity. The assessment of motility and viability was carried out by selecting concentrations of the treated samples in different random orders for each experiment.

In the second phase the effect of Catechol, at varying concentrations, was evaluated on the freshly collected semen sample to determine the effects on the sperm cell. The procedure followed was the same as in Phase I.

A minimum of 25 readings were taken for each sample from separate field. The experiment was repeated for control (*n* = 10), Phenol-hydroquinone (*n* = 12) treated samples, and Catechol (*n* = 12) treated samples. Samples with infections, pus cells, RBC, void volume, low count, and motility were excluded from the study.

Statistical analysis was carried out using Student's *t*-test and values were compared at 0.05% significance level. Values were expressed as mean ± standard error (SE).

Percent motility was determined according to the protocol in the WHO Laboratory Manual [12]. Percent viability (live : dead ratio) was determined using 0.1% Trypan Blue stain according to the procedure described by Talbot and Chacon [13]. Sperm nuclear DNA integrity was determined using the acridine orange fluorescence staining procedure described by Tejada et al. [14].

## 3. Results

Effects of benzene metabolites were evaluated on freshly collected semen samples of men of proven fertility and the toxicity indicator parameters studied were sperm motility, sperm viability, and nuclear DNA integrity.

### 3.1. Sperm Motility

The sperm motility was scored and it was observed that there was highly significant (*P* < 0.001) decrease in the percent sperm motility in Phase I after samples were exposed to Phenol-hydroquinone as compared to normal control samples. These results indicated an inverse correlation between concentrations of the aliquots and motility of sperm (Table 1).

In addition, under Phase II protocol the sperm motility was evaluated after serially diluting and exposing the semen sample at room temperature to Catechol of different concentrations. There was a significant (*P* < 0.01) decrease in the percent motility in Phase II as compared to normal control samples. Although significant alteration was found in the sperm motility of samples due to Catechol treatment (Phase II), the reduction in sperm motility was less than that observed with Phenol-hydroquinone under Phase I. Thus, these results indicate that Phenol-hydroquinone brought about a more highly significant reduction in sperm motility as compared to catechol (Tables 1 and 2).

### 3.2. Sperm Viability

Sperm viability was similarly evaluated after exposure of spermatozoa to varied concentrations of Phenol-hydroquinone *in vitro*. The study revealed that there was highly significant (*P* < 0.001) decrease in the percent sperm viability at 0.3 ppm concentration and significant (*P* < 0.01) decrease at 0.2 ppm concentration as compared to the control sample, whereas there was no significant effect of Phenol-hydroquinone on the sperm viability, at lower concentrations of 0.1 ppm and 0.05 ppm (Table 1).

Sperm viability was further evaluated after varied concentrations of Catechol. There was a highly significant (*P* < 0.001) decrease in percent sperm viability at concentrations of 0.3 ppm and 0.2 ppm as compared to the normal-control sample. Moreover, there was also a significant decrease in sperm viability (*P* < 0.01) at 0.1 ppm and 0.05 ppm concentrations after treatment with catechol (Table 2).

### 3.3. Sperm Nuclear DNA Integrity (Acridine Orange Fluorescence Double-Stranded DNA)

The percent green fluorescing spermatozoa, having intact, double-stranded DNA, were scored in sperm smears from normal and treated semen samples, and a significant decline was observed in the percent green fluorescing spermatozoa after treatment with both, phenol-hydroquinone and catechol, as compared to the untreated control samples. The number of spermatozoa with intact double stranded DNA was significantly lower (*P* < 0.001) in the treated (phenol-hydroquinone and catechol) samples as compared to normal-control group, at all concentrations tested (Table 3).

### 3.4. Effective Sperm Count

The effective sperm count, determined as the product of absolute sperm density and percent green fluorescing spermatozoa (divided by 100), showed a decline in both Phase I and Phase II experiments. Consequently, a highly significant decline was observed in the effective sperm count as compared to sperm density in samples exposed to both metabolites of benzene (Table 3).

**Figure 1:**
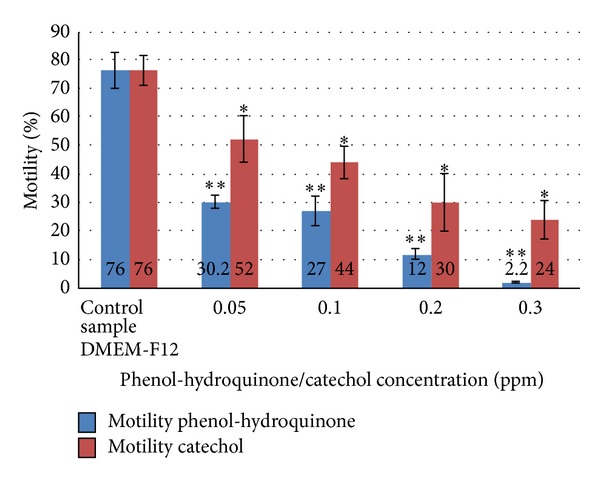
Comparison of motility between phenol-hydroquinone and catechol treated spermatozoa.

Figure 1 depicts the comparison of motility between Phenol-hydroquinone and Catechol treated spermatozoa.

**Figure 2:**
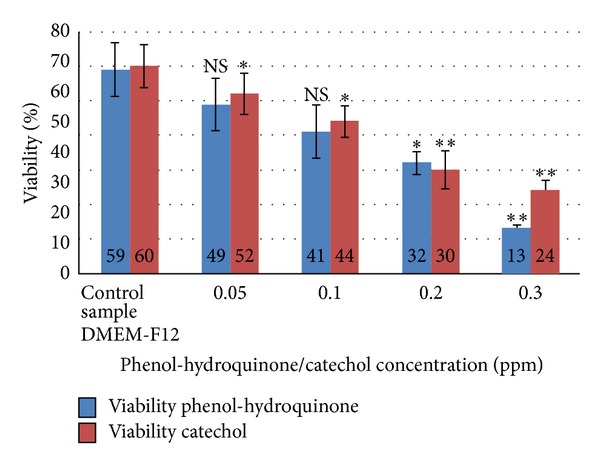
Comparison of viability between phenol-hydroquinone and catechol treated spermatozoa.

Figure 2 depicts the comparison of viability between Phenol-hydroquinone and Catechol treated spermatozoa.

## 4. Discussion

In recent years, individuals are increasingly being exposed to vapours of benzene, through paint, plastic, petroleum industries, fuel exhaust, and tobacco smoke. Benzene is readily absorbed following inhalation or oral exposures and is distributed rapidly in the body through blood circulation. The liver is the important site of benzene metabolism, which results in the production of several active metabolites. Sammett et al. [15] have shown that benzene toxicity is mainly associated with its metabolic products phenol-hydroquinone and catechol. There are several reports in the literature regarding the toxic influences of benzene exposure on various tissues [16–18].

Aiso et al. [19] have demonstrated elevated protein and cholesterol levels in liver after benzene exposure and have proved that the toxic impact is due to the action of a benzene derivative and metabolite. However, the toxicity associated with circulating levels of benzene metabolites, namely, phenol-hydroquinone and catechol, on sperm function has scarcely been reported in the literature.

Hence the present investigation was directed towards determining the effect of benzene metabolites, namely, phenol-hydroquinone and catechol, on the motility, viability, and nuclear integrity of the human spermatozoa.

From the results obtained in Phase I studies it was clear that exposure to phenol-hydroquinone caused a significant decline in both, sperm motility and viability. It has been well established that sperm motility depends largely upon the sustained generation of ATP via oxidative pathways. This metabolism requires several oxidative enzymes to be actively functional in the sperm midpiece.

Steiber et al. [20] have demonstrated that certain natural molecules could permeate the mitochondrial matrix and inhibit reactions within the complexes necessary for ATP generation. In addition, interference in the mitochondrial oxidative reactions conversely leads to production of excess reactive oxygen species (ROS) and free radicals. These in turn affect mechanism for normal metabolic functions and motility. Exposure to a phenol-hydroquinone microenvironment may therefore inhibit metabolically active enzymes, thus impeding ATP production, and in turn lowers sperm motility. Sperm motility has thus been considered as one of the most sensitive markers for sperm cytotoxic effects, as several drugs and toxic agents have been shown to cause altered sperm motility, due to decreased ATPase activity [8].

In addition sperm motility also depends on intact protein structures associated with contractility in the sperm tail [21, 22]. Phenol-hydroquinone has been shown to adversely affect protein structure and more specifically benzene metabolites have been proven to inhibit microtubular function [2].

It has also been reported that viability of the sperm cell is correlated with intact, functional, and semipermeable sperm plasma membranes. Loss of sperm membrane integrity has a direct bearing in lowering the survival of spermatozoa. The lowered viability due to phenol-hydroquinone exposure observed in this study could be explained as an outcome of loss of flagellar motility and metabolic incompetence as also observed by Eberhard et al. [23]. On the other hand, in the second phase of this study, Phase II, the exposure of human sperm *in vitro* to varied concentrations of catechol caused only insignificant changes in sperm motility and viability as compared to that observed on exposure to phenol-hydroquinone. Hence, exposure to Catechol appeared to have less toxic effects than that of Phenol-hydroquinone.

In addition, the present study also revealed that both metabolites of benzene caused significant denaturation of sperm nuclear DNA, since single-stranded, red fluorescing spermatozoa were found in a higher percentage after exposure. In a review Evenson et al. [11] stated that environmental stress, gene mutations, and chromosomal abnormalities disturbed the higher refined biochemical events that occurred during spermatogenesis, and this could ultimately lead to an abnormal chromatin structure that is incompatible with fertility. Chapman et al. [24] have confirmed that the phenol-hydroquinone induced toxicity in several tissues is based on endogenous bioactivation system and generation of free radicals. The Phenol-hydroquinone potentiated toxicity has also been reported to induce depressed bone marrow erythropoiesis [25]. Hence, exposure to phenol-hydroquinone *in vitro* could have resulted in generation of free radicals and altered membrane function, which is reflected by a decline in the motility, viability and loss of sperm nuclear DNA integrity.

In agreement with our results, an ASTDR Report [2] states that benzene's genotoxicity is derived primarily from its metabolites which inhibit topoisomerase and microtubule function and induce oxidative stress and DNA breaks. The metabolites of, benzene are known to be cytotoxic, affect nuclear DNA decrease cell proliferation [26], and have widely been associated with biochemical and metabolic alteration in both liver and kidney.

To conclude, the toxic impact of benzene exposure on sperm function is evident through the data obtained. However, further in-depth study is warranted to elucidate in particular the toxicity of benzene metabolites on other reproductive tissues and fertility.

## 5. Conclusion

The present study was aimed at determining the effects of benzene metabolites, namely, Phenol-hydroquinone and Catechol, on the motility, viability, and nuclear DNA integrity of the human sperm. The study revealed that Phenol-hydroquinone, at the varied concentrations tested *in vitro*, caused significant inhibition of sperm motility, viability, and loss of DNA integrity.

In comparison, Catechol had a less significant inhibitory effect on the sperm motility and viability *in vitro*. However, the data indicated that Catechol exposure had an adverse effect on the sperm nuclear DNA.

These benzene metabolites manifest their toxicity by generation of free radicals and reactive oxygen species. Phenol-hydroquinone also inhibits several enzymes and arrests microtubular contractility, thus directly inhibiting sperm motility and viability.

The action of these metabolites in generating free radicals could explain the induced toxicity. The study holds specific significance since man is exposed to vapours of benzene, through paint, plastic, petroleum industries, fuel exhaust, and tobacco smoke.

## Figures and Tables

**Table 1:** Table shows motility and viability after incubation with varied concentrations of phenol-hydroquinone.

Parameter	Control sample DMEM-F12	0.3 ppm	0.2 ppm	0.1 ppm	0.05 ppm
Motility	76 ± 6.5	2.2 ± 0.2**	12 ± 1.8**	27 ± 5.2**	30.2 ± 2.4**
Viability	59 ± 7.7	13 ± 1.0**	32 ± 3.4*	41 ± 7.8^NS^	49 ± 7.6^NS^

Values are mean ± SE: *n* = 10, 12; *P < 0.01, **P < 0.001; NS: not significant.

**Table 2:** Table shows motility and viability after incubation with varied concentrations of catechol.

Parameter	Control sample DMEM-F12	0.3 ppm	0.2 ppm	0.1 ppm	0.05 ppm
Motility	76 ± 5.3	40 ± 6.7*	51 ± 10*	57 ± 5.6*	67 ± 8.1*
Viability	60 ± 6.3	24 ± 3.1**	30 ± 5.5**	44 ± 4.6*	52 ± 5.9*

Values are mean ± SE; *P < 0.01, **P < 0.001.

**Table 3:** Table shows percent green/red fluorescing spermatozoa in normal and treated semen samples.

	Intact DS DNA (% green fluorescing)	Denatured SS DNA (% red fluorescing)	Effective DNA (%)
Normal	86.5 ± 9.2	14.5 ± 3.3	73.5
Phenol-hydroquinone treated (30 *μ*l)	47.6 ± 5.8**	63.3 ± 7.5**	40.5**
Catechol treated (30 *μ*l)	55.3 ± 8.4**	44.7 ± 6.1**	47**

Values are mean ± SE; **P < 0.001.

Effective sperm count = sperm density × % green fluorescing spermatozoa/100.
